# Differentiation between CSF Otorrhea and Rhinorrhea in an Obscure Case of Recurrent Meningitis

**Published:** 2014-04

**Authors:** Mohsen Rajati, Mohammad Mehdi Ghassemi, Mohammad Alipour, Mehdi Bakhshaee, Ayeh Shahabi, Masoud Naseri Sadr

**Affiliations:** 1 *Sinus and Surgical Endoscopic Research Center, Ghaem Hospital, Faculty of Medicine, Mashhad University of Medical Sciences, Mashhad, Iran.*; 2 *Department of Anesthesiology,Ghaem Hospital,Faculty of Medicine,Mashhad University of Medical Sciences,Mashhad,Iran.*; 3 *Department of Otorhinolaryngology, Ghaem Hospital,Faculty of Medicine,Mashhad University of Medical Sciences, Mashhad,Iran.*

**Keywords:** CSF, Fluorescein, Recurrent meningitis, Rhinorrhea.

## Abstract

**Introduction:**

Leakage of cerebrospinal fluid in the skull base may be accompanied with recurrent meningitis. The site of leakage may either be anterior (in the nose and paranasal sinuses) or posterior (in the temporal bone). Various imaging techniques can be used to precisely locate the point of leakage but despite all the advances in imaging techniques there are still some rare cases in which the surgeon can’t be sure on the management approach before the beginning of surgery.

**Case Report:**

In this article we present one of these cases; we used intrathecal fluorescein to locate the source of the leak and made the final decision on the operating table.

**Conclusion:**

Intrathecal fluorescein is helpful in locating the leakage in the ear or the nose in ambiguous cases.

## Introduction

Leakage of cerebrospinal fluid (CSF) through the temporal bone may cause various problems. The most common presentations are hearing loss due to middle ear effusion, persistent otorrhea after tympanostomy tube placement, and meningitis (1).Different imaging methods can be used to locate the site of leakage including CT, CT with intrathecal metrizamide, MRI, and radio-isotope studies. However, in some cases there may be questions about the precise point of leakage. Using intrathecal fluorescein during the surgery while implementing intracranial pressure-rising maneuvers is helpful in this regard (2).Once the defect is found, various repair methods can be employed such as middle fossa craniotomy, transcanal vestibular obliteration, and closure of the eustachian tube and the ear canal. Materials that can be used to seal the defects include muscle, fascia, fat, bone dust and chips, and pedicled flaps.

Herein we present the case of an eight-year-old boy with recurrent meningitis in whom the source of CSF leakage was in question so we used fluorescein to confirm the diagnosis. We also discuss the various repair methods

## Case Presentation

An eight-year-old boy was referred to our department due to recurrent meningitis. The parents reported the occurrence of a minor head trauma at the age of 15 months (falling from a stroller), and initiation of watery nasal discharge since then. The first episode of meningitis happened 3 months later and recurred 6 times in the next 4 years. At the age of 6 the boy was admitted to the neurosurgery department of a district hospital and, most probably because of the suspicious nasal discharge, a craniotomy was performed through a bicoronal approach to seal the assumed leakage at cribriform plate and the adjacent floor of the anterior fossa. In the 2 years following surgery the patient had no episodes of meningitis but he had had 2 attacks of meningitis in the past 6 months before being admitted to our department at a tertiary referral university hospital. 

**Fig. 1:**
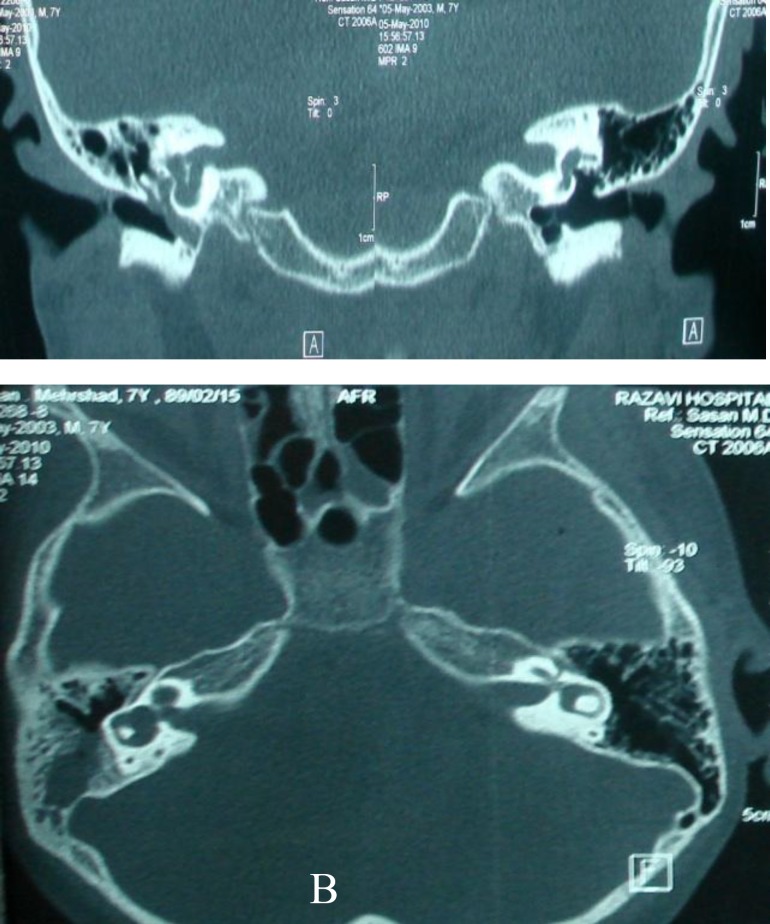
**A,B)** High-resolution CT images showing the deformity in the right inner ear

During the performance of a comprehensive otologic history the patient turned out to have hearing impairment in his right ear, which the parents maintained had happened after the first episode of meningitis. He had watery nasal discharge with an undetermined source (diagnostic tests couldn’t prove the discharge was CSF). The oscopic exam was normal but an audiogram showed right ear deafness and the tympanogram was type A on both sides. The high resolution CT revealed an anomaly in the inner ear on the right side (Figure 1). 

**Fig.2:**
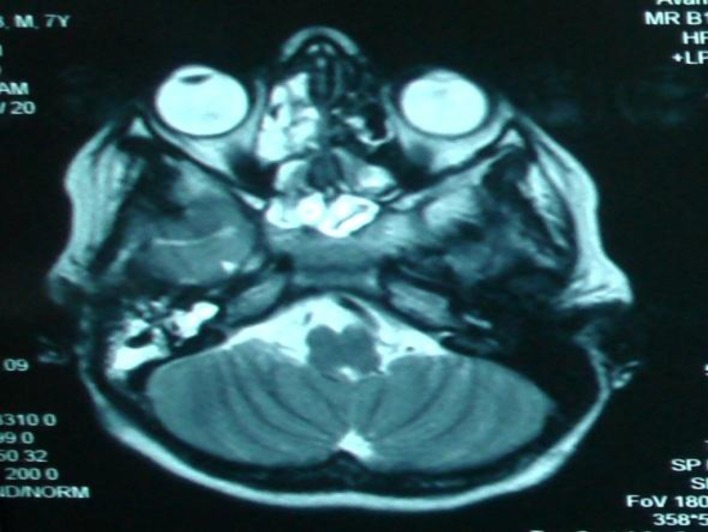
T2-MRI showing fluid in the ear and sinuses

The anomaly was most likely a mondini variant with associated defects in the lamina cribrosa and in the stapes footplate. An MRI was also completed which showed that the middle ear was partially filled with fluid and there was some collection of fluid in the paranasal sinuses (Figure 2).

**Fig. 3:**
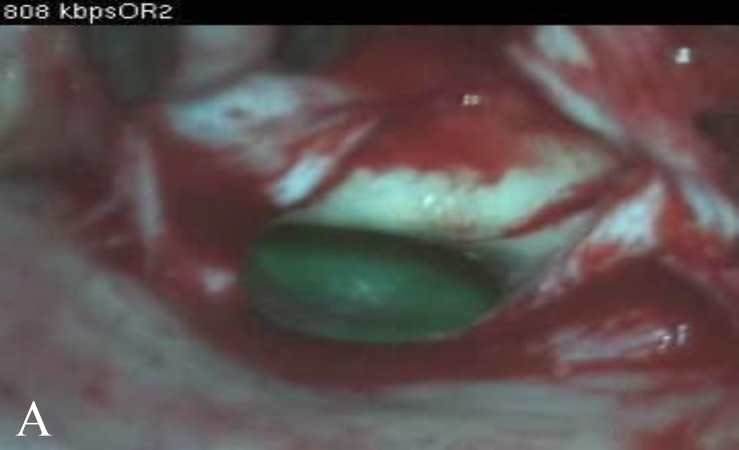
A view of the tympanic membrane after incising the posterior canal skin

Summing up our pre-operative information, we deduced that the inner ear was the culprit but the suspicious nasal discharge, temporary cessation of meningitis episodes after the craniotomy, obliterating anterior fossa floor, and fluid accumulation in the paranasal sinuses all challenged this presumption. Moreover, the small chance of simultaneous CSF otorrhea and rhinorrhea from two separate sources was another point we considered. So we decided to make the final diagnosis in the operating theatre. After inducing anesthesia we used an intrathecal dye; 20mg fluorescein diluted in 10 cc of the patient’s own CSF was injected into L3–L4 space. Initially, an endonasal endoscopy was performed. While implementing the maneuvers to increase intracranial pressure no dye was detected in the nose and in sinuses but a green-colored fluid was noticed in the nasopharynx coming out of eustachian tube orifice. Next we did an otomicroscopic examination and saw that the intact membrane had an accumulation of green fluid behind it (Figure 3).

An exploration of the middle ear was then performed and a defect in the footplate was detected. To correct the problem we did a stepedectomy and the vestibule was obliterated with an oversized muscle plug and then covered by a facial graft. The leakage stopped and the patient was discharged within 4 days. The patient was monitored for the following 18 months during which no significant events occurred.

## Discussion

Defects of the temporal bone that cause leakage of CSF can be classified into 2 groups based on etiology: *Secondary *defects, which occur with trauma, surgery (iatrogenic), or neoplasms invading the skull base, and the less common *primary (spontaneous)* forms. For the primary temporal bone defects 2 distinct populations of patients can be described, first, children with congenital malformations of the temporal bone, and second, adults with *no* known primary or secondary temporal bone disease (3). 

In adults with spontaneous leakage of CSF the location of the defect is most commonly found to be the floor of the middle fossa (tegmen tympani or tegmenmastoidi). Increased intracranial pressure may be the predisposing factor, or, in patients with normal intracranial pressure, remnants of the arachnoid granulations may play the causative role(4-5). On rare occasions this process may be bilateral(6).On the other hand, children with spontaneous CSF otorrhea most often have congenital inner ear anomalies associated with hearing loss. To have CSF otorrhea two abnormal passages are needed: one between the subarachnoid space and the inner ear (most commonly a defect in the bony plate between the vestibule and internal auditory canal) and the second between the middle and inner ear (the oval window being the most common route) (7). High-resolution CT scans and MRI are helpful to confirm the diagnosis. Vestibular dysplasia, defective lamina cribrosa, and a bulging oval window are useful diagnostic clues in CT scans (8). This type of leakage rarely occurs in adults and only a few cases have been reported in the literature (9,10).

Intraoperative determination of the site of CSF leakage is critical in successful closure of the leak. Finding the site during nasal endoscopic approaches is sometimes very difficult especially in anterior fossa leaks. Fluorescein has gained popularity in the indeterminate cases, both for diagnosis and evaluating the success of repair (2).A low dose of intrathecal fluorescein (less than 50 mg, diluted in CSF, and slowly admini- stered) after premedication with steroid and antihistamine agents is generally safe (2,11) but, because of rare compli- cations, it is advised that fluorescein be used cautiously (12). Fluorescein is also used intrao- peratively in endoscopic skull base surgery at the end of the procedure to identify any continuing leaks and this practice is strongly correlated with a reduction in post-op leakages (13). In the presented case we faced uncertainty in tracing the source of the leakage; the suspicious nasal discharge, temporary cessation of meningitis episodes after the craniotomy which sealed the anterior fossa floor, and fluid accumulation in the paranasal sinuses all challenged the presumption of otogenic infection. There are also some reports in the literature on simultaneous anterior and posterior fossa defects (14). So, we decided to make the final diagnosis in the operating theatre using intrathecal fluorescein.

When the source has been precisely detected, the next step is closing the fistula. There are different methods to do this (10). The recommended technique for this group of patients (those with hearing loss and inner ear anomalies) is stapedectomy and vestibular obliteration with muscle plug. The first authors' personal experience with this method is promising with no failure in the few cases that we have had in our center. However, some authors have reported failure rates of 30 to 60% and suggest the use of multiple-layered barriers in the vestibule (i.e., glue, muscle, and fascia, reinforced by a pedicle temporalis muscle flap) in addition to intra-operative continuous lumbar drainage (15). If this second method fails translabyrinthine IAC closure seems to be a wise option (7).

## Conclusion

Leakage of CSF is a condition in which lots of debate occurs over how to make the diagnosis, locate the site of leakage, and repair it. Patients with recurrent meningitis should always be thoroughly investigated in this regard. Imaging is of paramount importance and in cases where the source of the leak is unclear intrathecal fluorescein is helpful to get to the precise location of the leak. Finally, the surgeon should be ready to take advantage of different methods to successfully seal the flow of CSF.
